# Effects of different ozonized slurry‐ice treatments and superchilling storage (−1°C) on microbial spoilage of two important pelagic fish species

**DOI:** 10.1002/fsn3.486

**Published:** 2017-07-16

**Authors:** Gioacchino Bono, Charles Odilichukwu R. Okpala, Sergio Vitale, Vincenzo Ferrantelli, Annamaria Di Noto, Antonella Costa, Calogero Di Bella, Daniela Lo Monaco

**Affiliations:** ^1^ Istituto per l'Ambiente Marino Costiero – Consiglio Nazionale delle Ricerche (IAMC‐CNR) Mazara del Vallo Italy; ^2^ Istituto Zooprofilattico Sperimentale della Sicilia (IZSS) Palermo Italy; ^3^ Educare and Skills Training Network Middlesex United Kingdom

**Keywords:** antimicrobial effects, European anchovy, ozone treatment, sardine, slurry‐ice, superchilling storage

## Abstract

Combining different preservative treatments for improving quality and safety of fishery products increasingly receives global research attention. Consistent with this pursuit, the current research was undertaken to determine the effects of different ozonized slurry‐ice treatments and superchilling (−1°C) storage on microbial spoilage of European anchovy (*Eugraulis encrasicolus*) and sardine (*Sardina pilchardus*), which are two commercially important pelagic fish species. After the catch (within <5 hr) and at defined scheduled storage times, ozone has been discharged once on sardine (herein referred to as “One‐T”) and repeatedly/sequentially on European anchovy (herein referred to as “Seq‐T”). Microbiological analyses enumerated total viable count (TVC), *Bacillus* spp., *Enterobacteriaceae*,* Lactobacillus* spp., *Moraxella* spp., *Shewanella* spp., *Listeria monocytogenes* and *Pseudomonas* spp. Independent of potential antimicrobial effects of ozone during superchilling storage, no *Listeria* spp., *Shewanella* spp., *Moraxella* spp., and *Bacillus* spp. were found in all processed samples. While *Enterobacteriaceae* and *Lactobacillus* spp. were detected at below 1 log cfu/g, both TVC and *Pseudomonas* spp. proliferated at different rates throughout superchilling storage. The repeated ozone‐treated (“Seq‐T”) showed lower TVC and *Pseudomonas* spp. values compared with one‐time treated (“One‐T”) slurry‐iced and control samples. Thus, combined slurry‐ice and superchilling storage at Seq‐T produced improved antimicrobial activity over One‐T application. Largely, ozonized slurry‐ice outcomes/results appear promising thanks to superchilling storage.

## 1 INTRODUCTION

Among the key aims of innovative food processing methods is the increasing pursuit for food safety. Some notable seafood processing methods include modified atmosphere packaging, ozone treatment, high pressure processing, irradiation as well as active and intelligent packaging (Bono & Badalucco, 2012; Bono et al., 2016; Bono, Okpala, Badalucco, Milisenda, & Vitale, 2017; Chen, Chang, & Ing, 1987; O'Donnell, Tiwari, Cullen, & Rice, 2012; Okpala (2014a,b); Okpala (2015a,b); Okpala, Bono, Abdulkadir, & Madumelu, 2015; Okpala, Bono, & Gancitano, 2015; Okpala, Bono, Falsome et al. 2016; Okpala, Bono, Geraci, et al., 2016; Okpala, Bono, Cannizzaro, & Jereb, 2016; Okpala & Bono, 2016; Sivertsvik, 2003; Zhang et al., 2011). According to US Food and Drug Administration (FDA), ozone treatment is a “Generally Recognized As Safe (GRAS)” process (Chawla, Bell, Janes, & Pollet, 2006; O'Donnell et al., 2012; Okpala, 2016a,b; Okpala, 2017a,2017c, 2017d, 2017e). Postharvest ozone treatments inhibit microbial growth in such fishery products as farmed turbot (Campos, Losada, Rodríguez, Aubourg, & Barros‐Veláquez, 2006), Pacific white shrimp (Okpala, 2014a,b, 2015a,b; Okpala, 2016a,b; Okpala, 2017a,2017c, 2017d, 2017e) rockfish (Kötters et al., 1997) as well as shucked mussel (Manousaridis et al., 2005) under different (cold) storage conditions. Free radical activity of molecular ozone can decrease growth (up to inactivation) of specific microbiological species like *Escherichia coli, Salmonella typhimurium*,* Staphylococcus aureus*,* Vibrio parahaemolyticus*, and *Vibrio cholerae* (O'Donnell et al., 2012). Besides ozone containing water considered as nondestructive and reliable means of fishery products’ processing, the efficacy of ozone treatment would depend on such factors as pH of medium, posttreatment residual activity, quantity of ozone applied, and temperature (Naito & Takahara, 2008; O'Donnell et al., 2012; Okpala, 2014b). As previous researchers have considered the capacity of ozone to affect lipid oxidation and sensorial properties of fishery products (Campos, Rodríguez, Losada, Aubourg, & Barros‐Veláquez, 2005; Campos et al., 2006; Chen et al., 1987; Kötters et al., 1997; Manousaridis et al., 2005; O'Donnell et al., 2012; Okpala, 2017a,2017c, 2017d, 2017e), the microbiological aspects still require further investigations.

Fundamentally, microbial proliferation quickens as immune system that controls microbiological contents of fishery products collapses during postharvest. The harvest method, geo‐location, catch season, pH, period time between harvest and consumption, salinity and temperature with storage are able to influence the microbial proliferation in fishery products. Alongside other pertinent non‐microbiological constituents responsible for fish deterioration, microbiological activities inevitably contribute to limit cold/refrigerated postharvest shelf storage (Cadun, Kışla, & Çaklı, 2008; Lu, 2009; Okpala, 2014a; Okpala, Choo, & Dykes, 2014). Besides, microbiological assessment of fishery products under aerobic cold storage conditions respectively considers either 10^6^ and/or 10^7^ cfu/g as upper acceptable limits for both psychrotrophic (e.g., *Pseudomonas* spp.) and mesophilic bacteria such as H_2_S‐producing types (e.g., *Shewanella* spp.) (Bensid, Ucar, Bendeddouche, & Özogul, 2014; Özogul, Polat, & Özogul, 2004). Slurry‐ice that constitute ice‐water suspension and sub‐zero temperature of up to −1.5°C, is among refrigeration candidates with a strong preservative potential for the fishery industry, which on one hand, is owed to the physical feature of ice‐water suspension that increasingly facilitates the chilling rate (through rapid heat exchange within fish muscle) and on the other, the spherical microparticle nature that helps to reduce the skin/surface damage (Kılınc, Caklı, Cadun, Dıncer, & Tolasa, 2007; Múgica, Barros‐Velázquez, Miranda, & Aubourg, 2008). In parts of Northern Europe, awareness among fisheries companies about the benefits of applying slurry‐ice primarily to improve shelf quality appears to be on the increase, for example, fresh Cod species. However, this is much less among fishing communities of Mediterranean basin especially small highly perishable pelagic fishery products like European anchovy (*Eugraulis encrasicolus*), sardine (*Sardina pilchardus*), and so on. In particular, postharvest preservation challenge of rapid microbial degradation of these fish species directly affects the product/shelf quality (Bensid et al., 2014; Campos et al., 2005).

Whilst slurry‐ice chilled storage applied to fishery products has been increasingly studied (Kılınc et al., 2007; Lin, Deng, Huang, & Guo, 2016; Múgica et al., 2008; Rodriguez, Carriles, & Aubourg, 2010; Zhang, Deng, & Wang, 2015), relevant information specific to ozonized slurry‐ice to reduce microbiological contents in small pelagic fish (Campos et al., 2005, 2006) appears inadequate. For instance, Campos et al. (2005, 2006) applied one time (postharvest) ozone treatment (biphasic water ratio mix of 40% ice – 60% water) and concentration discharge (of range between 0.17 to 0.2 mg/L), then slurry‐ice (−1.5°C), and refrigerated storage of 2°C to perishable pelagic sardine (*Sardina pilchardus*) and farmed turbot (*Psetta maxima*). These authors’ reported such treatments with promising preservative potentials in reducing microbiological contents such as aerobic mesophiles, psychrotrophic bacteria and lactose‐fermenting *Enterobacteriacea*e. In this context, there is need to formulate research questions that would help pave a way to supplement these previous studies, which had previously applied one‐time postharvest ozone treatment of up to 0.2 mg/L concentration. Notably, the choice and use of research questions in science, according to Okpala (2017b), has been considered as very useful in the definition, collection and reporting of relevant information. Consequently, the doing of each research endeavor ought to be clearly defined with adequate and robust visualization.

Specifically, this current study was goaled to utilize a higher ozone concentration discharge of 0.3 mg/L, with the aim to determine the effects of either one‐time postharvest ozone treatment (“**One‐T**”) and or repeated ozone treatments (“**Seq‐T**”) during superchilling storage (−1°C) on the microbial spoilage of European anchovy (*E. encrasicolus*) and sardine (*S. pilchardus*).

## 2 MATERIALS AND METHODS

### 2.1 Overview of experimental schedule

The experimental schedule of ozone treatment, slurry‐ice and superchilling storage of anchovy and sardine, followed by subsequent analyses is given in Table 1.

**Table 1:** Experimental schedule of ozone treatment, slurry‐ice and superchilling storage of anchovy and sardine samples, and subsequent microbial analyses

Day	Samples and analysis				Storage conditions
schedule	Anchovy		Sardine	Control (all samples)	
	Lot 1	Lot 2	(Lot 3)	(Lots 4 and 5)^a^	Slurry‐ice + superchilling
Day 0	“One‐T”	“Seq‐T”	“One‐T”	N.R.	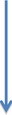
Day 1	Microbial analysis	Microbial analysis	Microbial analysis	Microbial analysis	
Day 3	N.R.	“Seq‐T”	N.R.	N.R.	
Day 4	Microbial analysis	Microbial analysis	Microbial analysis	Microbial analysis	
Day 7	N.R.	“Seq‐T”	N.R.	N.R.	
Day 8	Microbial analysis	Microbial analysis	Microbial analysis	Microbial analysis	
Day 11	N.R.	N.R.	Microbial analysis	Microbial analysis^b^	

“One‐T”, Ozone treatment performed once at Day 0; Seq‐T, Ozone treatment performed sequentially at scheduled times during storage period. N.R. No record of activity/analysis.

a Control samples for the respective lots of Anchovy and Sardine.

b Microbial analysis performed for control of Sardine at Day 11 only.

### 2.2 Sample collection up to treatment protocol

Directly onboard a “purse seine” fishing boat that traditionally are not equipped with slurry‐ice machine, European anchovy (*E. encrasicolus*) and sardine (*S. pilchardus*) caught in the Strait of Sicily (Central Mediterranean) were dipped in 1:1 mixture of seawater and traditional flake‐ice previously prepared in order to smoothen (by the water thermic effect) the characteristic damaging capability of this kind of ice. Upon arrival at the land, both fish species were dripped and delivered to the laboratory of IAMC CNR within <5 hr. The quantity of the fish species were as follows: ~10 kg of sardine and ~15 kg of European anchovy.

At the laboratory, the chilling procedure previously described by Múgica et al. (2008) with some modifications applied. It involved a prototype facility (La Felsinea Electroindustriali S.r.L, Italy) that has been used to produce the slurry ice mix, which contained 50% ice–50% marine water. Previously described by Bono and Badalucco (2012) and with slight modifications, the ozone treatment method has been applied as follows: The first ~ 10 kg of sardine has been divided in two equal lots. One lot of ~ 5 kg labeled “**One‐T**” was dipped in ~ 10 L slurry‐ice (w/v of 1:2 with marine water) to ensure a rapid cooling of the core part of muscle. Subsequently, it was treated with 0.3 mg L^−1^ of ozone using SGC11 O_3_ generator (Lenntech B.V., Rotterdamseveg, The Netherlands), and gently agitated for 15 min. At the next, maintaining in an appropriate amount of slurry‐ice, the sample was kept at superchilling storage (−1°C) until required for analyses. Then, a second lot of ~ 5 kg dipped as abovementioned that had no ozone treatment and subject to only slurry‐ice and superchilling storage (−1°C) served as “**Control**”.

On the other hand and given the very high perishability of European anchovy (as compared to sardine) likely owed to some specific spoilage organisms (SSO), which are largely known to be directly involved in chemical and sensory changes during chilling storage, a more structured antimicrobial processing – based on repeated ozone treatment “**Seq‐T**” during storage time, has been performed for this specific fish species of this current study. Using ~15 kg of European anchovy samples divided into three equal lots, one lot of ~ 5 kg labeled “**One‐T**” was dipped in ~ 10 L slurry‐ice (w/v of 1:2 with marine water) to ensure a rapid cooling the core part of muscle. Subsequently, it was treated with 0.3 mg L^−1^ of ozone using SGC11 O_3_ generator (Lenntech B.V., Rotterdamseveg, The Netherlands), and gently agitated for 15 min. Then, maintaining in an appropriate amount of slurry‐ice, the sample was kept at superchilling storage (−1°C) until required for analyses. Secondly, a second lot of 5 kg labeled “**Seq‐T**” dipped as abovementioned was treated with 0.3 mg L^−1^ of ozone sequentially at times of days 0 (day of harvest/arrival on land of samples), 3, and 7. And all the ozone‐processed European anchovy samples have been kept under slurry‐ice and superchilling storage (−1°C) prior to analysis at designated day(s). Thirdly, another lot of ~ 5 kg dipped as abovementioned that had no ozone treatment and subject to only slurry‐ice and superchilling storage (−1°C) served as “**Control**”.

When all treatments had completed, the samples continued with storage under superchilling condition (−1°C). Concurrently, microbiological quantification scheduled up to day 8 and 11 were performed on European anchovy and sardine, respectively. When required, the marine water slurry‐ice has been supplemented during storage periods.

### 2.3 Microbiological analysis

All processed and control samples was enumerated for total viable count (TVC), *Bacillus* spp., *Enterobacteriaceae*,* Lactobacillus* spp., *Moraxella* spp., *Shewanella* spp., *Listeria monocytogenes* and *Pseudomonas* spp. using standard references. All media employed for all microbiological enumeration was procured from Oxoid Ltd, UK. Beyond TVC, the colonies were isolated on selective media and in most cases required further confirmation and identification using the required biochemical tests. At the predetermined time intervals, aseptically obtained sample flesh (*E. encrasicolus* and *S. pilchardus*) (~30 g) for all treatments and independently has been homogenized (~120 s) (Stomacher Lab‐Blender 400, PBI International, Milan, Italy) in 270 mL of (w/v) sterilized saline peptone water with subsequent tenfold serial dilutions.

Specifically, the total viable count (TVC)/mesophilic aerobic flora was determined according to the ISO 4833‐1: 2013 method and enumerated on plate count agar (PCA), with aerobic incubation at 30°C for 72 hr. *Bacillus* spp. was determined according to the ISO 7932: 2004 method and enumerated on MYP (Mannitol Egg Yolk Polymyxin) Agar with incubation at 30°C for 48 hr, thereafter confirmation of suspect colonies seeded on blood agar plates that were incubated at 37°C for 24 hr. *Enterobacteriaceae* spp. was determined according to the ISO 21528‐ 2: 2004 method and enumerated on Violet Red Bile Glucose Agar (VRBGA) followed by incubation at 37°C for 24 hr, which the suspect colonies were confirmed following subculture in Nutrient Agar plates at 37°C for 24 hr followed by oxidase and fermentation tests. *Lactobacillus* spp. was determined according to the ISO 15214: 1998 method and enumerated on MRS Agar (DeMan, Rogosa, Sharpe), already thermostatically controlled at ~47°C, followed by incubation at 30°C for 72 hr, which the suspect colonies that appeared as large clear colonies confirmed by Gram stain and Oxidase test. *Moraxella* spp. and *Shewanella* spp. have been determined according to the method described by Koziñska and Pêkala (2004) and enumerated on TSA Agar, Aeromonas Agar and MacConkey Agar followed by incubation at 27°C for 24–48 h, where after suspect colonies have been placed on Nutrient Agar for 24 h, before confirmation using oxidase test, then identification with API20E kit (BioMérieux). *L. monocytogenes* was detected through ELFA (Enzyme Linked Fluorescent Assay) method, using miniVIDAS^®^ instrument (BioMérieux), according to the AFNOR BIO 12/11‐03/04 procedure and UNI EN ISO 11290‐1, 2005: the method provides the preventive incubation of 25 g of sample in 225 mL of primary (Half Fraser Broth) and then secondary enrichment (Fraser Broth by adding the respective supplements) for 24 h at 30°C. According to UNI EN ISO 11290‐2: 2005 method, *L. monocytogenes* enumeration required enrichment Half Fraser Broth, after incubation for 1 hr at 20°C, was inoculated into ALOA agar (Agar Listeria Ottaviani and Agosti) thereafter incubated at 37°C for 48 hr. According to ISO 13720: 2010 method, *Pseudomonas* spp was determined and enumerated using CFC (Cetrimide, Fucidin, Cephalosporin) Agar with incubation at 25°C for 48 hr, prior to confirmation and identification through oxidase and API20E tests (BioMérieux). Independently and for all treatments, the microbiological assays have been triplicated and results presented in logarithm of colony forming units per gram (log cfu/g).

### 2.4 Statistical analysis

**Figure 1:**
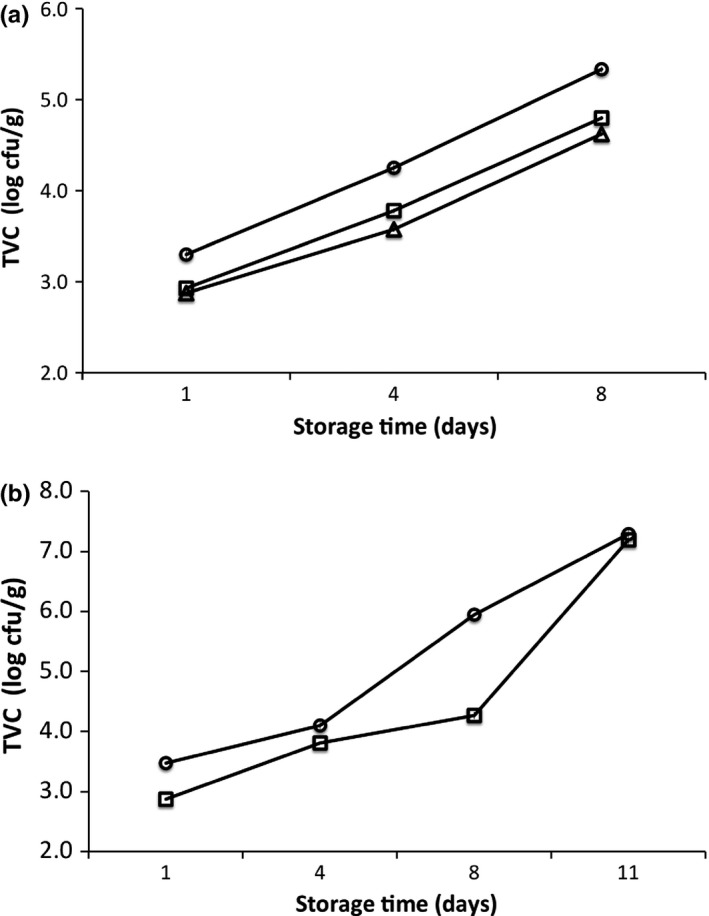
(a&b): Evolution of total viable count (TVC) of ozonized slurry‐ice and control European anchovy (a) and sardine (b) samples during superchilling storage time (days). Symbols: (Circle) = Control; (Block) =  “One‐T” (One‐time ozone treatment); (Triangle) = “Seq” (Sequential ozone treatment)

**Figure 2:**
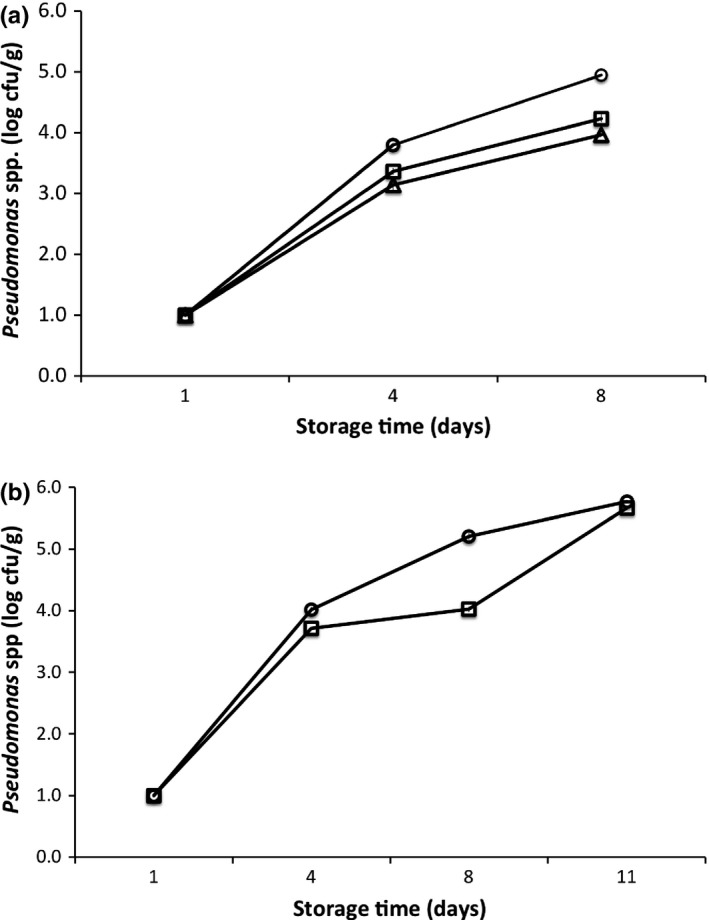
(a&b). Evolution of *Pseudomonas* spp. of ozonized slurry‐ice and control European anchovy (a) and sardine (b) samples during superchilling storage time (days). Symbols: (Circle) = Control; (Block) = “One‐T” (One‐time ozone treatment); (Triangle) = “Seq” (Sequential ozone treatment)

One‐way analysis of variance (ANOVA) was performed to establish treatment effects with storage time. To compare between treatments, Sample‐T test has been applied. Mean differences were resolved post hoc using Tukey's honest significant difference (HSD) test. In all cases, the probability level of *p *<* *.05 was considered as statistically significant. In Figures 1, 2, error bars (denoting the standard deviation (SD) of replicated measurements using different samples) appear smaller than the symbols. Minitab Express^TM^ software (version 1.3.0 for Mac, Minitab Ltd., Coventry CV3 2TE, UK) was employed to do the statistical analysis.

## 3 RESULTS AND DISCUSSION

This current study has been designed to establish if and to what degree the ozonized slurry‐ice treatment combining slurry‐ice and superchilling storage would produce antimicrobial effects on these two commercially important pelagic fish species (European anchovy and sardine). This has been performed through the application of two different ozone treatments, namely: one‐time “**One‐T**” and repeated/sequential “**Seq‐T**”. For emphasis, this latter/sequential approach (applied to the anchovy alone) aimed for any additional inhibitory effect of ozone on specific spoilage organisms (SSO) for example, *Pseudomonas* spp., which in abovementioned delicate fish species according to Gram (1995) are more directly involved in the production of deteriorative (chemical and sensory) attributes. Further, *Pseudomonas* spp., widely seen as ubiquitous and linked to its metabolic diversity, thrives at low temperatures to cause deteriorative spoilage (Gennari & Dragotto, 1992).

The TVC and *Pseudomonas* spp. continuously proliferated (*p* < .05) with superchilling storage even as ozone slurry‐ice treatments were applied to both European anchovy and sardine samples (Figure 1 and 2). A closer look at the Figure 1b as a start, the TVC in the sardine samples can be seen to exhibit rather visible lag phase compared with its somewhat absence at the other treatments. Specifically, this somewhat absence of lag phase seem apparent at microbiological proliferations (both for TVC and *Pseudomonas* spp.) of other previous studies concerning the same or similar pelagic fishery products stored at between 3 and 4°C (Bensid et al., 2014; Erkan & Özden, 2008; Pons‐Sánchez‐Cascado, Veciana‐Nogués, Bover‐Cid, Mariné‐Font, & Vidal‐Carou, 2006; Stamatis & Arkoudelos, 2007). Comparing both Figures 1 and 2, the ozonized slurry‐iced approach can be seen to further inhibit microbial proliferation compared with the control (slurry‐ice only). Specific to anchovy samples with storage, the TVC comparing “**Seq‐T**” and “**One‐T**” showed statistical differences (*p *<* *.0001) both at days 4 (“Seq” = ~3.58 log cfu/g; “One‐T” = ~3.78 log cfu/g; *F* = 149.52, R.sq.(adj.)=93.4%) and 8 (“Seq” = ~ 4.62 log cfu/g; “One‐T” = 4.80 log cfu/g; *F* = 672.95, R.sq. (adj.)=99.4%). Furthermore TVC of both “**Seq‐T**” and “**One‐T**” had less values than control, with “**Seq‐T**” noticeably less compared to “**One‐T**” (*p *<* *.05) (Fig. 1a). The result suggests that “**Seq‐T**” approach supplemented with superchilling storage can be a promising candidate to decrease further the microbial proliferation of such delicate fishery products. Particularly, this result, on one hand, appears to strengthen the antimicrobial capacity of ozone treatment, and on the other, opens new scenarios regarding other potential uses even when applied at low dosage/quantities with storage period/time. Indeed, the microbiological effect of “**Seq‐T**” approach of this study can be likened to another sequential ozone‐treated ice‐stored crustacean product that had less microbial numbers compared to control at later stages of storage time compared with the earlier (Okpala, 2014a). With respect to sardine samples (Figure 1b) and somewhat different from the (above‐described) anchovy ones, the “**One‐T**” showed significantly lower TVC values at days 1 (*p *<* *.0001, *F* = 1643.57, R.sq (adj) = 99.7%), 4 (*p *=* *.0058, *F* = 28.83, R.sq(adj.) = 84.8%), 8 (*p *<* *.0001, *F* = 2298.47, R.sq. (adj.) = 99.8%) but not at day 11 (*p *>* *.05) compared to slurry iced (control) ones. The result further indicates that the “one time” postharvest ozone treatment might not necessarily guarantee consistent/continued antimicrobial effects and for long period of time for the reason that the shelf time of ozone‐processed samples in this particular scene of this study lasted for only but slightly after day 8.

Comparing European anchovy (Figure 1a) and sardine (Figure 1b) and regardless of the applied ozone treatment, the detected *Pseudomonas* spp. values resembled (*p *>* *.05) but only at day 1. However, subsequent days showed *Pseudomonas* spp. with varying significant effects (*p *<* *.05) and with lower values at ozonized samples compared to non‐ozonized ones. Specific to anchovy, the “**Seq‐T**” treated samples showed *Pseudomonas* spp. less at days 4 (~3.14 log cfu/g) (*p *=* *.02, F = 13.81, R.sq. (adj.) = 71.9%) and 8 (~3.96 log cfu/g) (*p *=* *.0014, *F* = 61.51, R.sq (adj.) = 92.34%) compared with the “**One‐T**” ones. And specific to sardine, although the “**One‐T**” treated samples showed significantly lower *Pseudomonas* spp. values (*p *=* *.0008, F = 82.37, R.sq.(adj.) = 94.2%) by day 4, it showed increasing tendencies by day 8 (*p *<* *.0001, F = 764.12, R.sq.(adj.) = 99.4%) but not so at day 11 (*p* > .05) compared with the slurry‐iced ones (control). This result appears to connect with abovementioned observation about TVC in sardine (Figure 1b) where ozone maintained some inhibition effect on TVC at least up to day 8 of storage time. Further, some increases in *Pseudomonas* spp seem apparent and at a faster rate over TVC especially between days 1 and 4 (Figures 1 and 2b). Despite such proliferation, ozone treatment can be said to show some reducing effects on *Pseudomonas* spp. and at the subsequent days. At the end of superchilling (−1°C) storage in ozonized slurry‐ice (for emphasis: 50% ice–50% marine water), both *Pseudomonas* spp and TVC values of anchovy and sardine of between ~ 3.96 and 5.67 and 4.62 and 7.19 log cfu/g were noticeably below those of non‐ozonized slurry‐ice (i.e., control) of between ~ 4.95 and 5.77 and 5.34 and 7.29 log cfu/g, respectively. In addition, not only being gram‐negative and halophilic/salt‐tolerant, *Pseudomonas* spp. can thrive in harsh conditions given their hardy cell walls (Van Eldere, 2003), which might be contributing in facilitating some resistance to the ozone treatment of this current study.

In view that either 10^6^ or 10^7^ cfu/g indicated respective upper acceptable limits for psychrotrophic and mesophilic bacteria (Bensid et al., 2014; Özogul et al., 2004), the microbial data outcome at end of storage of current study should be considered within the acceptable limit(s). Moreover, this tested superchilling approach method might have contributed to make the slurry‐ice effective with storage time. Superchilling storage stands as potential candidate to facilitate the ozone decomposition mechanism to quickly reach inhibition phase a bit earlier. Following report of O'Donnell et al. (2012), the inhibition phase of ozone decomposition mechanism might actuate the scavenging activity of bicarbonate ions to facilitate inactivation of microbial cells. And probably at such biochemical milieu, hydroxyl radicals get consumed without further regeneration of superoxide radical ion. In this context, ozone treatment improves the degree of oxidative destruction of microbial cell wall to cause cell lysis, which can result in the cell contents to leak into its surrounding medium (O'Donnell et al., 2012). For the reason that the collapse of immune system of fishery product remains irreversible at postharvest and progressing with microbiological proliferation up to spoilage even under cold/iced storage, any variations in the detected/observed microbiological proliferation would continually depend on both innate microbial entities and cultivation environment (Okpala et al., 2014; Özogul & Balikci, 2013; Venugopal, 2005). Therefore, how microbiological proliferation develops in untreated pelagic fishery product such as European anchovy / sardine should never be underestimated even under slurry‐iced situation(s).

The degree of attachment of microbial entities on food surface significantly can influence the bactericidal effects of ozone. Thus, the latter can be attributed to the unstable allotrope of oxygen, which makes its oxidizing properties very reliable (O'Donnell et al., 2012). In the ozone and non‐ozone processed samples of this current study, the enumeration of TVC, *Enterobacteriaceae* spp., *Lactobacillus* spp., *Listeria* spp., *Pseudomonas* spp., *Shewanella* spp., *Moraxella* spp. as well as *Bacillus* spp were performed. Regardless of the potential effects of ozone treatment, no *Listeria* spp., *Shewanella* spp., *Moraxella* spp., and *Bacillus* spp. were detected in any processed samples. On the other hand, both *Enterobacteriaceae* and *Lactobacillus* spp. remained below the 1 log cfu/g mark. To the best of our knowledge, this present study appears to be the first to test the combined effects of slurry‐ice and superchilling storage on proliferation of the above bacterial species in these two important pelagic fish species. Besides, Campos et al. (2005) seems to be the only relevant work that applied slurry‐ice (−1.5°C) and refrigerated storage of 2°C to perishable pelagic sardine. However, existent relevant literatures (Erkan & Özden, 2008; Pons‐Sánchez‐Cascado et al., 2006; Stamatis & Arkoudelos, 2007) that reported above bacterial species specific to the studied pelagic fish species have been subject to other cold ice/refrigerated storage conditions of between 3 and 4°C. In particular, Stamatis and Arkoudelos (2007) reported initial low *Enterobacteriaceae* spp. value (~1 log cfu/g) in sardine stored at 3°C that thereafter had seemingly clear increase up to ~ 4 log cfu/g around the mid part of storage time. This was different for *Shewanella putrefaciens* and *Pseudomonas* spp. that continuously proliferated to surpass 7 log cfu/g at that same study. Erkan and Özden (2008) and Pons‐Sánchez‐Cascado et al. (2006) obtained different increasing bacterial proliferation trends at storage condition of ~ 4°C on *Enterobacteriaceae* and *Pseudomonas* spp. in sardine and European anchovy, respectively.

**Figure 3:**
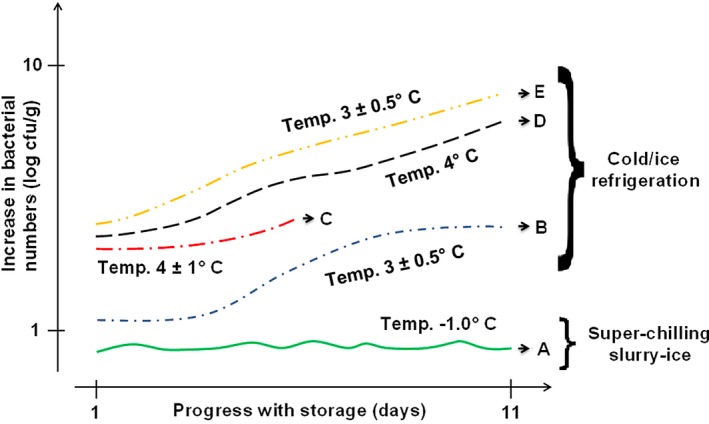
Schematic illustration of estimated proliferation increment(s)/trend(s) of some (relevant/specific) bacterial species detected in (some) small pelagic fishery products as affected by different storage temperatures and times (days). Specifically, “A” refers to bacterial species of *Enterobacteriaceae* and *Lactobacillus* spp. in European anchovy and sardine samples of current study under slurry‐iced superchilling storage condition; “B” refers to bacterial species of *Enterobacteriaceae* spp. in refrigerated sardine samples as reported by Stamatis and Arkoudelos (2007); “C” refers to bacterial species of *Enterobacteriaceae* spp. and *Pseudomonas* spp. in refrigerated sardine kept under ice (shorter period) as reported by Erkan and Özden (2008); “D” refers to bacterial species of *Enterobacteriaceae* spp. and *Pseudomonas* spp. in ice‐stored/refrigerated anchovy kept under flake ice as reported by Pons‐Sánchez‐Cascado et al. (2006); “E” refers to bacterial species of *Shewanella putrefaciens* and *Pseudomonas* spp. in refrigerated sardine samples as reported by Stamatis and Arkoudelos (2007)

Before we conclude, it is worthwhile to contextualize the slurry‐iced superchilling storage of this current study with those of ice‐stored/refrigerated storage previously reported and both scenarios are concerned with related/relevant small pelagic fish species. We do this via the schematic illustration of estimated bacterial proliferation trends of abovementioned bacterial species detected in some small pelagic fishery products as affected by different storage temperatures, here depicted in Figure 3. Given that these abovementioned bacterial species and associated pelagic species are akin to our current study, there are equally comparable. Clearly, the bacterial load values of below 1 log cfu/g under slurry‐ice superchilling storage of this current study (referred herein as trend “A”) is below those obtained at works of Erkan and Özden (2008) (referred herein as trend ‘C’), Pons‐Sánchez‐Cascado et al. (2006) (referred herein as trend “D”), as well as Stamatis and Arkoudelos (2007) (referred herein as trends “B” and “E”). The reduction of storage temperature(s) from ~ 3°C (refrigeration) up to −1°C (i.e., slurry‐ice and superchilling) seems the only deducible factor that can explain the inhibition of *Enterobacteriaceae* spp. proliferation, the latter widely responsible for production of spoilage off‐odors. From this visual schematization, the beneficial effect of superchilling storage (if supplemented with slurry‐ice) can be deduced, compared with traditional refrigeration approach widely employed in the fishery supply chain.

## 4 CONCLUSIONS

From the data provided by this current study, the ozonized slurry ice combined with superchilling storage shows improved antimicrobial potential on both European anchovy and sardine samples. Specifically, the “**Seq‐T**” performed better to reduce the microbial load of ozonized‐slurry iced approach compared with the “**One‐T**”. The results of ozonized slurry‐iced approach appear promising thanks to superchilling storage. To the best of our knowledge, this is the first report that investigated the effects of (different) ozonized slurry‐ice and superchilling (−1°C) storage treatment conditions on microbial spoilage of two important pelagic fishery species (specifically, European anchovy and sardine). Given that the antimicrobial effects of ozonized slurry ice samples appears promising under superchilling storage (−1°C), future studies should be directed to define the best/optimized ozone treatment (e.g., low ozone dosage continuously applied with storage time or medium dosage sequentially or yet high dosage one time), which, not only accords with corresponding (potential) oxidative effects (in particular on unsaturated lipid component{s}) of fishery flesh product quality, but also, the acceptable safe level for on‐board staff handling. And this should be performed so as to generate additional yet robust data to supplement the current information. Other such characteristic attributes as physicochemical and sensory properties of similar/other related pelagic species submitted to (different) combinations of slurry ice/ozone composition/concentrations equally deserves attention in future studies.

## CONFLICT OF INTEREST

None declared.
